# Data-Driven Structural Health Monitoring: Leveraging Amplitude-Aware Permutation Entropy of Time Series Model Residuals for Nonlinear Damage Diagnosis

**DOI:** 10.3390/s24020505

**Published:** 2024-01-13

**Authors:** Xuan Zhang, Luyu Li, Gaoqiang Qu

**Affiliations:** 1 School of Civil Engineering, Dalian University of Technology, Dalian 116024, China; dgzx@mail.dlut.edu.cn (X.Z.); qugq@mail.dlut.edu.cn (G.Q.); 2 State Key Laboratory of Coastal and Offshore Engineering, Dalian University of Technology, Dalian 116024, China

**Keywords:** structural health monitoring, nonlinear damage, data-driven, time series models, amplitude-aware permutation entropy, distributed sensor systems

## Abstract

In structural health monitoring (SHM), most current methods and techniques are based on the assumption of linear models and linear damage. However, the damage in real engineering structures is more characterized by nonlinear behavior, including the appearance of cracks and the loosening of bolts. To solve the structural nonlinear damage diagnosis problem more effectively, this study combines the autoregressive (AR) model and amplitude-aware permutation entropy (AAPE) to propose a data-driven damage detection method. First, an AR model is built for the acceleration data from each structure sensor in the baseline state, including determining the model order using a modified iterative method based on the Bayesian information criterion (BIC) and calculating the model coefficients. Subsequently, in the testing phase, the residuals of the AR model are extracted as damage-sensitive features (DSFs), and the AAPE is calculated as a damage classifier to diagnose the nonlinear damage. Numerical simulation of a six-story building model and experimental data from a three-story frame structure at the Los Alamos Laboratory are utilized to illustrate the effectiveness of the proposed methodology. In addition, to demonstrate the advantages of the present method, we analyzed AAPE in comparison with other advanced univariate damage classifiers. The numerical and experimental results demonstrate the proposed method’s advantages in detecting and localizing minor damage. Moreover, this method is applicable to distributed sensor monitoring systems.

## 1. Introduction

During the operation of civil engineering structures, various factors such as environmental conditions, loads, material aging, and structural fatigue may affect the structure’s performance, leading to gradual degradation or damage. If these damages are not detected and repaired promptly, the consequences can be catastrophic. Therefore, structural health monitoring (SHM) is increasingly crucial in civil engineering [1,2,3]. Its primary objective is to accurately identify, locate, and quantify the damage within structures, enabling timely maintenance interventions and extending their service life. In this process, developing accurate and reliable damage diagnosis techniques is paramount.

Currently, SHM techniques are divided into two categories: data-driven and model-driven [3,4,5]. Data-driven methods extract damage-sensitive features (DSFs) from measurement data and perform statistical decisions based on damage classifiers [6,7]. These DSFs, such as waveform spectra and modal properties, need to be sensitive to damage but insensitive to environmental variability factors. On the other hand, if a physical model is established, these features can be linked to the physical information of the structure, enabling the application of model-driven methods to determine the location and extent of damage [8,9]. However, the current field of SHM typically assumes the studied objects to be linear systems. The development of these techniques focuses on the linear discounting of stiffness, which assumes a representation of the dynamic response before and after damage based on a linear structural model [10]. In reality, damage is often accompanied by nonlinear behavior, such as crack opening and closing, bolt loosening, and delamination of structural adhesive layer materials under operational loads [11,12]. The presence of these nonlinear phenomena poses challenges to linear model-based methods, while some data-driven DSFs still hold potential for application [13,14,15,16]. Therefore, data-driven methods based on statistical pattern recognition have garnered significant attention in addressing nonlinear damage detection.

In data-driven approaches, time series model analysis is an advantageous tool for structural nonlinear damage feature extraction [3,17]. Both the coefficients and model residuals of a time series model contain rich damage information, and they are typically used as DSFs. While methods based on model coefficients are effective, the advantage of using model residuals is avoiding re-determining model orders and parameter estimates [18]. In addition, it has been shown that the statistical features of the model residuals, such as the magnitude, gradually increase with increasing damage. Therefore, in recent years, researchers have proposed several effective damage classifiers based on the statistical characteristics of the model residuals, and most of them have been validated in nonlinear benchmark structural data in the Los Alamos National Laboratory. These damage classifiers include some simpler univariate distance techniques, such as the nonlinear damage identification method using the residuals’ standard deviation of a vector autoregressive (VAR) model proposed by Mattson and Pandit [19]. The outcomes demonstrated that their method for damage identification performed better than conventional approaches based on higher-order moments and could provide information about the damage’s location. Chegeni et al. [20] developed the coefficient relative error index (CRI) and the relative residual index (RRI) as damage classifiers using autoregressive (AR) models. Their research demonstrated that residual-based classifiers produce better results than coefficient-based classifiers. Entezami et al. [21] proposed a residual reliability criterion (RRC) damage classifier for the DSFs of AR model residuals and verified that it outperforms some classical techniques through comparative analysis. To address the limitations of high-dimensional DSFs, several hybrid distance methods using model residuals have emerged. For instance, Entezami et al. [22] proposed a hybrid distance method of the autoregressive-autoregressive with exogenous input (AR-ARX) model residuals combining partitioned Kullback–Leibler divergence (PKLD) and mahalanobis distance (MD). This approach solves the problem of using high-dimensional damage features in statistical decision-making and enables early damage detection under various environmental and operational conditions. Daneshvar et al. [23] employed Kullback–Leibler divergence (KLD) and the residual relative error (RRE) to dimensionality reduction of AR model residual and then combined the reduced features with the MD for damage diagnosis. In a subsequent study, Daneshvar et al. [24] used a Gaussian mixture model to dimensionally reduce the AR-ARX model residuals and combined them with the MD for early damage diagnosis of cable-stayed bridges.

Some traditional time series model approaches are similarly based on the statistical characterization of the model residuals. While these methods may not apply to unsupervised learning, they are effective for nonlinear damages. For example, Chen et al. [25] used the AR/ARCH model in economics for nonlinear damage detection in structures and proposed the use of a second-order variance index (SOVI) as a damage indicator. Zuo and Guo [26] developed an AR model for structural response acceleration data and proposed a damage identification method based on the residual Kullback–Leibler distance of the AR model to identify the nonlinear damage location of the structure more effectively. However, most of the previous studies have focused on the statistical characteristics of the model residuals, emphasizing the capability of the proposed damage classifiers in structural damage diagnosis and localization. In reality, the low level of nonlinear damage is often the source of the progressive deterioration of large civil engineering structures. Damage classifiers should exhibit higher sensitivity to minor damages to provide a more accurate early-stage damage diagnosis. Therefore, it is necessary to explore different damage classifiers based on model residuals to investigate their sensitivity in low-level nonlinear damage diagnosis.

The concept of information entropy originated from information theory. It is a method used to quantify the complexity and irregularity of signals and systems. Generally, higher entropy values indicate greater uncertainty in the signal, while lower entropy values indicate stronger regularity. Damage alters the response signal of a structure, leading to changes in the predictability of dynamic responses. Therefore, a damaged system exhibits a different level of complexity compared to a baseline system. Over the past few decades, several entropy-based methods have been introduced in the field of SHM, such as sample entropy [27,28], fuzzy entropy [29], permutation entropy [30], and spectral entropy [31]. Information theory-based methods have emerged as a new approach to novelty detection [32].

Among these entropy methods, permutation entropy (PE) is theoretically simpler, relatively more robust to observations and dynamic noise, and has faster computational speed. These characteristics make PE an attractive tool for handling a wide range of practical signal applications [33]. For instance, Soofi et al. [30] employed PE and inverse transmissibility function to investigate a damage diagnosis method based solely on entropy metrics. Li et al. [34] utilized multiscale PE to develop a novel method for detecting metal fatigue crack propagation. PE can also be used in the process of damage diagnosis based on the residuals of time series models. Unlike the statistical characterization of the model residuals, the PE can detect changes in the complexity of the residual series. Chen et al. [35] were the first to apply PE to feature extraction of residuals in time series models and analyze the influence of coupled disturbances between operational variability and structurally nonlinear damage on residual PE. Their research showed that residual PE is a robust nonlinear damage classifier that is not easily affected by environmental factors. However, PE also has some limitations. First, when symbolizing the signal, only the relative order of the sequence is taken into account, which leads to the loss of amplitude information. Secondly, PE does not address the influence of equal amplitudes within each embedding vector. Therefore, Azami et al. [36] proposed amplitude-aware permutation entropy (AAPE) to compensate for the shortcomings of PE. Their study showed that AAPE, which takes into account the mean and difference of the sample amplitudes, is more sensitive to the signal’s amplitude characteristics than PE, and is a powerful tool for segmenting signals and detecting spikes. At present, AAPE has been used for image classification [37], feature extraction [38,39,40], and has been widely used in the mechanical field, especially in rotating machinery fault diagnosis [41,42].

This paper presents a data-driven nonlinear damage diagnosis method based on AR models and AAPE. Given the high sensitivity to nonlinear damage, the AR model is employed to fit the baseline data, and the model residuals for the unknown states are extracted as DSFs [43]. Due to higher sensitivity to signal complexity and amplitude, AAPE is used as a novel damage classifier of model residuals for diagnosing and localizing nonlinear damage. The efficacy of the proposed method is substantiated through numerical simulations involving a six-story building model and experimental data obtained from a three-story frame structure at the Los Alamos Laboratory. In addition, the damage classifier based on the residual AAPE of the AR model is compared with some existing univariate classifiers in terms of diagnosis of minor damage. The results demonstrate that the proposed method is more sensitive to minor damage, and accurate damage localization can be performed even in the case of minor damage.

## 2. Feature Extraction Using AR Models

### 2.1. AR Models

The AR models are a class of statistical models commonly used in time series analysis and forecasting, indicating that the value of the current moment can be a linear combination of the values of the previous *p* moments. Its mathematical expression is:

(1) $$X_{t}=c+\phi_{1}X_{t-1}+\phi_{2}X_{t-2}+\ldots +\phi_{p}X_{t-p}+\varepsilon_{t}$$

where $X_{t}$ is the observed value at time *t*, *c* is a constant term, $\phi_{1},\phi_{2},\cdots ,\phi_{p}$ are the model coefficients, and $\varepsilon_{t}$ is a white noise error. AR models are commonly used for feature extraction in signal processing because they can extract periodic features of signals and are sensitive to nonlinear features [3].

### 2.2. Testing AR Model Applicability

Although the AR model applies to vibration data for most structures, it is still necessary to validate its applicability before using the AR model for feature extraction. The applicability of the AR model can usually be determined by the Box–Jenkins graphical method or the numerical method of the Leybourne-McCabe (LMC) hypothesis test [24]. The Box–Jenkins method examines the time series data’s autocorrelation function (ACF) and partial autocorrelation function (PACF) plots. When the ACF curve shows a trailing tail, and the PACF curve shows a truncated tail, the AR model can be considered appropriate; otherwise, the autoregressive moving average model needs to be considered. However, the graphical approach can become complex when dealing with multiple data sets. In such cases, the LMC hypothesis test can provide a numerical assessment for selecting the model type. It can be used for automated model selection with multiple sets of data. These numerical values include the *p*-value, *c*-value, and the LMC statistic at a significance level α. If the *p*-value > significance level α or the LMC statistic < *c*-value, it can be concluded that the sequence conforms to an AR model.

### 2.3. Determining AR Model Order

The selection of an appropriate model order is critical for accurately extracting DSFs. From a statistical perspective, the non-correlation of the residual samples is an essential criterion for determining the accuracy and appropriateness of the model. We used an iterative approach based on the Ljung–Box Q-test (LBQ) test to determine the model order [21,44]. Like the LMC hypothesis test, the LBQ test provides some numerical decision-making values. When the *p*-value is greater than the significance level, or the test statistic is less than the *c*-value, the null hypothesis holds, implying that the model residuals are uncorrelated. Therefore, as the model order gradually increases, the order of uncorrelated residuals obtained by the LBQ test is considered the finally determined model order.

Since iteration of the model order starting from zeros can be time-consuming, we can use the Bayesian information criterion (BIC) to determine the model order and then iterate through the LBQ test. The BIC considers the trade-off between goodness-of-fit and model complexity, and it aims to select a model that can fit the data better while avoiding overfitting. The BIC is calculated as follows:

(2) $$BIC=-2\ln\left(L_{\max}\right)+a\ln\left(n\right)$$

where $\ln\left(L_{\max}\right)$ is the value of the log-likelihood function of the model, indicating the extent of the model’s fit to the data, *a* is the number of parameters estimated in the model, and *n* is the number of samples. The Bayesian criterion-based model order iteration method can significantly reduce the time required for computation.

### 2.4. Extracting AR Residual Features

The coefficients of the AR model are reliable DSFs that can be obtained by the Euler–Walker method or the least squares method [26]. However, DSFs based on AR model residuals do not require recalculation of model coefficients and are more compatible with unsupervised learning for damage detection. Therefore, researchers prefer to use model residuals as DSFs. The principle lies in the fact that when damage occurs, the actual response of the structure will deviate from the predicted response of the baseline model, thus allowing the detection of anomalies. The model residual is calculated below and is the difference between the actual response $x_{t}$ and the baseline model predicted response ${\hat{x}}_{t}$.

(3) $$\varepsilon_{t}=x_{t}-{\hat{x}}_{t}$$

Once residual features are obtained, several damage classifiers can be used to detect the occurrence of anomalies.

## 3. Nonlinear Damage Diagnosis Based on Amplitude-Aware Permutation Entropy of AR Model Residuals

### 3.1. Nonlinear Damage with Bilinear Stiffness

In real structures, nonlinear stiffness and damping effects make the damage exhibit nonlinear characteristics [45]. For example, some steel beams or columns will exhibit some elastic stiffness when initially loaded. Once a fatigue crack occurs, the structure develops different stiffnesses during the crack opening and closing phases. Although the structure may exhibit linear behavior at different stages, the rapid opening and closing of cracks under sustained loading makes the stiffness vary continuously during vibration. Therefore, localized cracks in vibration can be considered as time-varying nonlinear systems. This fatigue crack with variable stiffness is a typical nonlinear damage during continuous vibration of the structure. The bilinear stiffness models have been widely used to simulate crack-induced nonlinear damage in chain structures [10,46]:

(4) $$k_{i}\left[x_{i}\left(t\right)\right]=\left\{\begin{array}{cc} k_{i} & \;\mathrm{when}\;x_{i}\left(t\right)-x_{i-1}\left(t\right)\leq d\\ \left(1-\beta \right)k_{i} & \;\mathrm{when}\;x_{i}\left(t\right)-x_{i-1}\left(t\right)>d \end{array}\right.\;\left(i=1,\ldots ,n;x_{0}\left(t\right)=0\right)$$

where the stiffness $k_{i}\left[x_{i}\left(t\right)\right]$ of the *i*th degree of freedom changes in response to the opening and closing of cracks. When the crack is closed, the stiffness is $k_{i}$; when the crack is open, the stiffness is reduced to an β fraction of the original. Here, β represents the stiffness reduction coefficient caused by fatigue cracks. The opening and closing of the crack are related to the relative displacement of this degree of freedom. $x_{i}\left(t\right)$ and $x_{i-1}\left(t\right)$ represent the lateral displacements of the *i*th and (i−1)th degrees of freedom. The parameter *d* has been used to model the severity of nonlinear behavior [47]. It is worth noting that when i=1, $x_{i-1}\left(t\right)=x_{0}\left(t\right)=0$, indicating that the displacement of the structure’s foundation is zero.

### 3.2. Damage Classifiers Using Statistical Features

For comparison with damage classifiers based on amplitude-aware permutation entropy, we introduce a Kullback–Leibler divergence (KLD) [23] univariate damage classifier that utilizes statistical features of the model residuals. KLD describes an asymmetric measure of two probability distributions and can represent the deviations that occur in the probability distribution of a signal. Assuming that $P_{x}$ and $P_{z}$ are the PDFs of the *n*-dimensional baseline state residual feature vector $\varepsilon_{u}$ and the damage state residual feature vector $\varepsilon_{d}$, respectively, the KLD can be represented by the following formula:

(5) $$KLD=\sum_{l=1}^{n_{r}}P_{x}\left(l\right)\log\frac{P_{x}\left(l\right)}{P_{z}\left(l\right)}$$

where $n_{r}$ is the number of probability samples. Since both $P_{x}$ and $P_{z}$ are probability density functions, the KLD always gives a positive value to represent the distance. When KLD is close to zero it means that the probability distributions of the two signals are close to each other, and when KLD is greater than zero, the two probability distributions have been shifted. Sensor locations with higher KLD typically indicate regions where structural damage is likely to occur.

### 3.3. Damage Classifiers Using Amplitude-Aware Permutation Entropy

#### 3.3.1. Permutation Entropy

Permutation entropy (PE) is a metric employed for analyzing the complexity and randomness of time series data. For a time series consisting of *N* data points, denoted as X=[x(1),x(2),x(3),…,x(N)], the computation of PE begins with phase space reconstruction.

By embedding dimension *m* and time delay τ, the data can be reconstructed as the matrix $X^{m,\tau }$:

(6) $$X^{m,\tau }=\left[\begin{array}{c} X_{1}^{m,\tau }\\ X_{2}^{m,\tau }\\ X_{i}^{m,\tau }\\ \cdots \\ X_{K}^{m,\tau } \end{array}\right]=\left[\begin{array}{cccc} x(1) & x(1+\tau ) & \cdots & x(1+(m-1)\tau )\\ x(2) & x(2+\tau ) & \cdots & x(2+(m-1)\tau )\\ x(i) & x(i+\tau ) & \cdots & x(i+(m-1)\tau )\\ \cdots & \cdots & \cdots & \cdots \\ x(K) & x(K+\tau ) & \cdots & x(K+(m-1)\tau ) \end{array}\right]$$

The $X^{m,\tau }$ matrix divides the data into *K* subsequences and K=N−(m−1)τ. Since the embedding dimension is *m*, each row has *m* elements.

**Figure 1:**
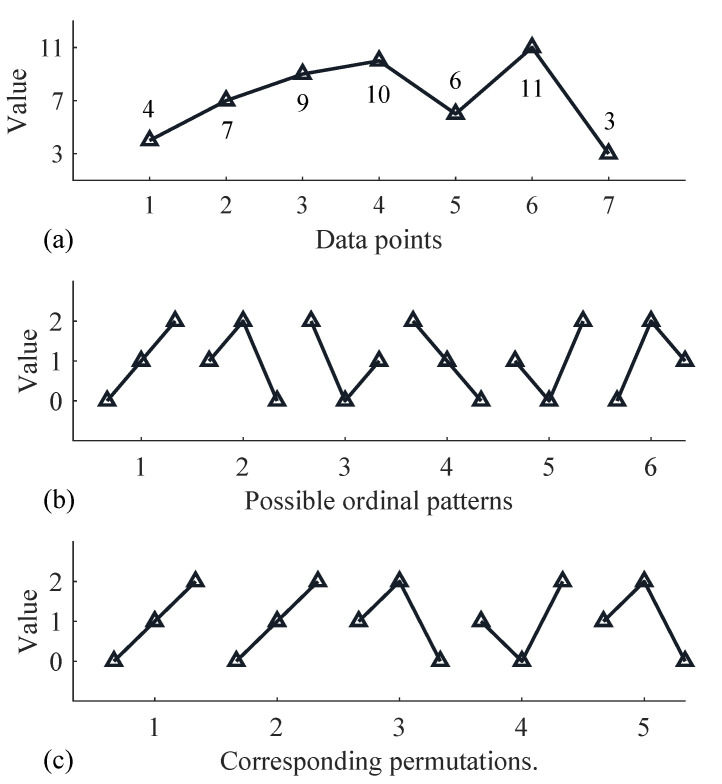
An example calculation of permutations, including (**a**) original data points, (**b**) possible ordinal patterns, (**c**) corresponding permutations.

After partitioning into subsequences, the *m*-dimensional vectors in $X^{m,\tau }$ matrix are mapped to unique permutations $\pi_{i}$, thereby capturing the ordinal ranking of the data. For an *m*-dimensional sequence, there are *m*! possible ordinal patterns $\pi_{i}$ that could occur. However, in practice, only a subset of these may actually occur. We denote the actual number of occurring permutations as J(J<m!). Figure 1 takes the sequence [4,7,9,10,6,11,3] as an example and shows the possible ordinal patterns when m=3, τ=1, as well as the permutations corresponding to this sequence. At this point, m!=6, and J=3.

Define the number of occurrences of the permutation index $\pi_{i}$ as $v\left(\pi_{j}\right)$; then, the probability $p_{j}$ that $\pi_{j}$ occurs is:

(7) $$p_{j}=\frac{v\left(\pi_{j}\right)}{K}$$

Thus, PE can be obtained by the following equation:

(8) $$PE=-\sum_{j=1}^{J}p_{j}\log_{2}p_{j}$$

#### 3.3.2. Amplitude-Aware Permutation Entropy

Amplitude-aware permutation entropy (AAPE) is an extension and improvement of traditional PE. Traditional PE only considers the order of permutation patterns, while it overlooks the amplitude information of subsequences. For instance, in Figure 1, both subsequences [4 7 9] and [7 9 10] are mapped to the same pattern π = [0 1 2], contributing equally to the final value of PE. This indicates that traditional PE is not sensitive to variations in signal amplitudes. In contrast, AAPE takes into account the amplitude information of data points within subsequences, making it more suitable for handling time series data that possess amplitude information.

In AAPE computation, the first step is similar to the PE method, which involves reconstructing phase space. However, AAPE defines the probability of the occurrence of the *j*th ordinal patterns as follows:

(9) $${\bar{p}}_{j}=\frac{\sum_{p}\omega \left(X_{p}^{m,\tau }\;\mathrm{has}\;\mathrm{type}\;\pi_{j}\right)}{\sum_{q=1}^{N-(m-1)\tau }\omega \left(X_{q}^{m,\tau }\right)}$$

For a certain subsequence $X_{i}^{m,\tau }=\left\{x\left(i\right),x\left(i+\tau \right),\ldots ,x\left(i+\left(m-1\right)\tau \right)\right\},1\leq i\leq K$, $\omega \left(X_{i}^{m,\tau }\right)$ is defined as follows:

(10) $$\omega \left(X_{i}^{m,\tau }\right)=\frac{\lambda }{m}\sum_{k=1}^{m}\left|x\left(i+\left(k-1\right)\tau \right)\right|+\frac{1-\lambda }{m-1}\sum_{k=2}^{m}\left|x\left(i+\left(k-1\right)\tau \right)-x\left(i+\left(k-2\right)\tau \right)\right|$$

In Equation (10), λ represents the adjustment coefficient between the subsequence sample difference and mean, with values ranging between [0,1]. As λ approaches zero, the difference holds a higher weight relative to the mean. Conversely, as λ approaches 1, the mean carries a higher weight relative to the difference. Once the probability of sorting is determined, subsequent steps are identical to the PE method. Therefore, AAPE can be calculated using the following formula.

(11) $$AAPE=-\sum_{j=1}^{J}{\bar{p}}_{j}\log_{2}{\bar{p}}_{j}$$

As the damage intensifies, the PE and AAPE of the model’s residuals tend to decrease. However, the correspondence between AAPE and the degree of damage is entirely opposite to that of a univariate classifier using KLD. To facilitate comparison with the univariate classifier using statistical features, we applied a max-normalization to PE and AAPE. Specifically, we identified the maximum value from the data and then subtracted all other data using the maximum value. Through this transformation, the entropy-based classifier tends to increase with the damage worsening.

#### 3.3.3. Selection of AAPE Parameters

Before employing AAPE as a damage classifier, it is essential to determine the parameters associated with AAPE. Typically, the embedding dimension *m* of AAPE is selected within the range of 3 to 7. This selection is motivated by the consideration that, when *m* is less than 3, the number of permutation states becomes insufficient for effectively extracting the main features of the data sequence. Conversely, if *m* is excessively large, although the value of the AAPE may be more reliable, it leads to a significant increase in computation time. Azami et al. [36] suggest that m! ≪ *N*, where *N* represents the time series length, and the sampling rate τ can be chosen between 1 or 2. In this study, we choose *m* to be 5 and τ to be 1 for illustration. In the comparative study of the subsequent cases, the PE parameters are chosen the same as in AAPE. As for the adjustment coefficients, we choose λ=0.5 because the sensitivity of AAPE to amplitude and frequency is the most significant in this case.

#### 3.3.4. Unsupervised Damage Diagnostic Process

**Figure 2:**
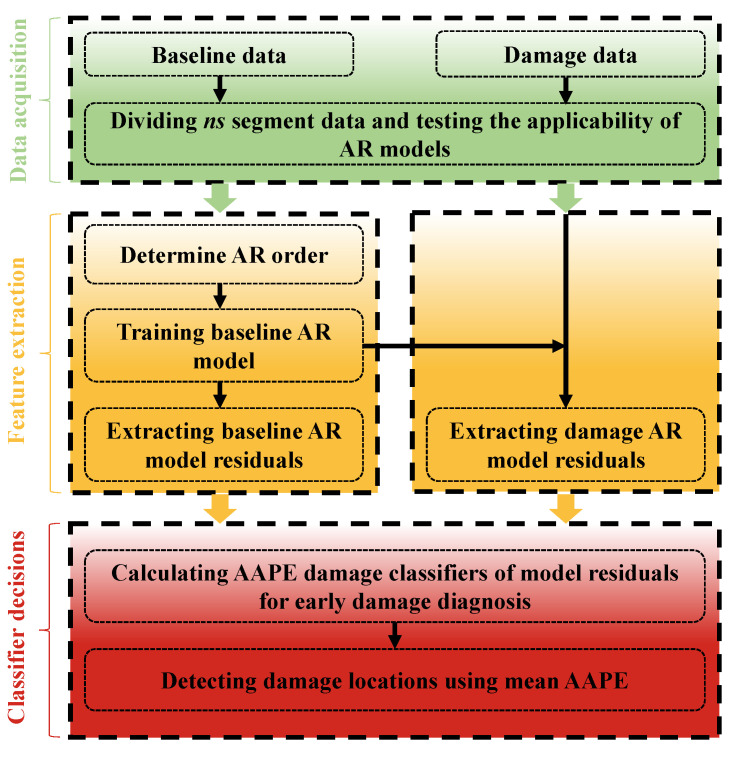
Damage diagnosis process of the proposed method.

The structural data corresponding to the various damage states are divided into ns segments to facilitate statistical representation of the residual features of the AR model. The mean values of the AR model coefficients trained in the healthy state are used to build the baseline AR model. AR model residuals extracted from the dataset with multiple damage states are used to diagnose damage for the AAPE damage classifier. The damage identification process of the proposed method is as follows. For simplicity, Figure 2 shows the flowchart of the proposed method.

Step 1: The responses from individual sensors of the structure, under varying states, are partitioned into ns segments. Subsequently, the Box–Jenkins and LMC hypothesis testing methods are employed to scrutinize whether the structural responses adhere to the AR model.

Step 2: During the baseline state, an iterative approach grounded in the BIC is applied to ascertain the AR model order for the response of each sensor. The ensuing step involves computing the average of model coefficients and establishing a baseline AR model for each sensor.

Step 3: The model residuals of the ns segment response for each sensor are calculated at different damage states, and then the AAPE is calculated to detect structural anomalies.

Step 4: The mean AAPE value is computed for multiple sets of segments corresponding to different states for each sensor. Subsequently, normalization is applied to identify locations of nonlinear damage.

## 4. Numerical Case

### 4.1. Introduction to the Six-Story Building Model

**Figure 3:**
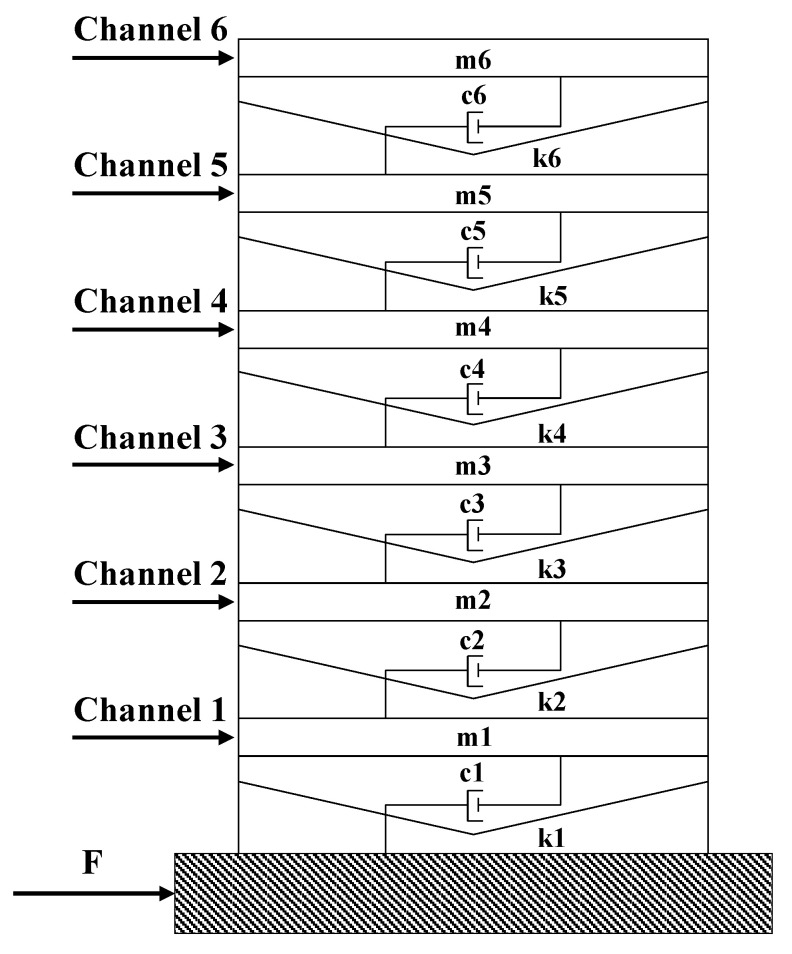
Numerical model of a six-story building subjected to nonlinear damage.

To validate the superiority of the AAPE damage classifier, a simulated six-story building model subjected to nonlinear damage, as shown in Figure 3, is employed in this study. The structure’s mass is set to $M_{i}=1$ kg per floor, the stiffness value of each floor is $K_{i}=23$ kN/m, and the damping ratio of the structure is 0.05. The interlayer stiffness-to-mass ratios are inspired by Entezami et al. [48]. Gaussian white noise is applied at the bottom to simulate the random seismic loads on the structure. In the six-story building model, a bilinear stiffness as shown in Equation (4) is introduced to simulate damage similar to fatigue cracks in the columns between neighboring floors. The structural elastoplastic behavior is not considered. As shown in Table 1, to evaluate the performance of the damage classifier, nonlinear damage is introduced gradually at the third and sixth floors, with the severity of the damage gradually increasing from minor damage.

**Figure 4:**
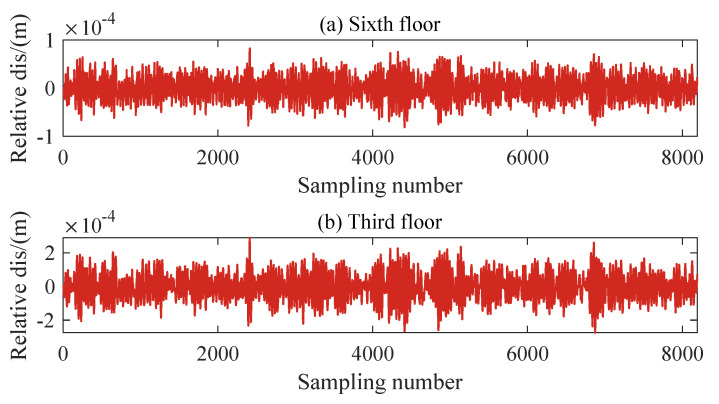
Relative displacement of the (**a**) sixth, and (**b**) third floors.

Similar to the nonlinear damage modeled in the Los Alamos three-story frame structure in the experimental case, the stiffness reduction factor β in Equation (4) is a fixed value set to 0.1. The extent of damage is determined by the parameter *d* and the relative displacement of the damaged floors, as shown in Figure 4. This allows for a more favorable simulation of minor damage. When *d* is relatively large, the structure remains healthy and does not exhibit nonlinear behavior. As *d* decreases gradually, the probability of nonlinear behavior increases, and the corresponding severity increases. The acceleration sensors are distributed and placed on each floor. For each damage condition, 409,600 data points were recorded for each channel to be used for subsequent damage diagnostic analysis, with a sampling frequency of 200 Hz. The nonlinear response of the structure is simulated through Simulink with the fixed-step Runge–Kutta method [46]. Additionally, white noise with an energy ratio of 2% is added to the structural response to simulate signal noise.

### 4.2. Nonlinear Damage Identification Process and Results

**Figure 5:**
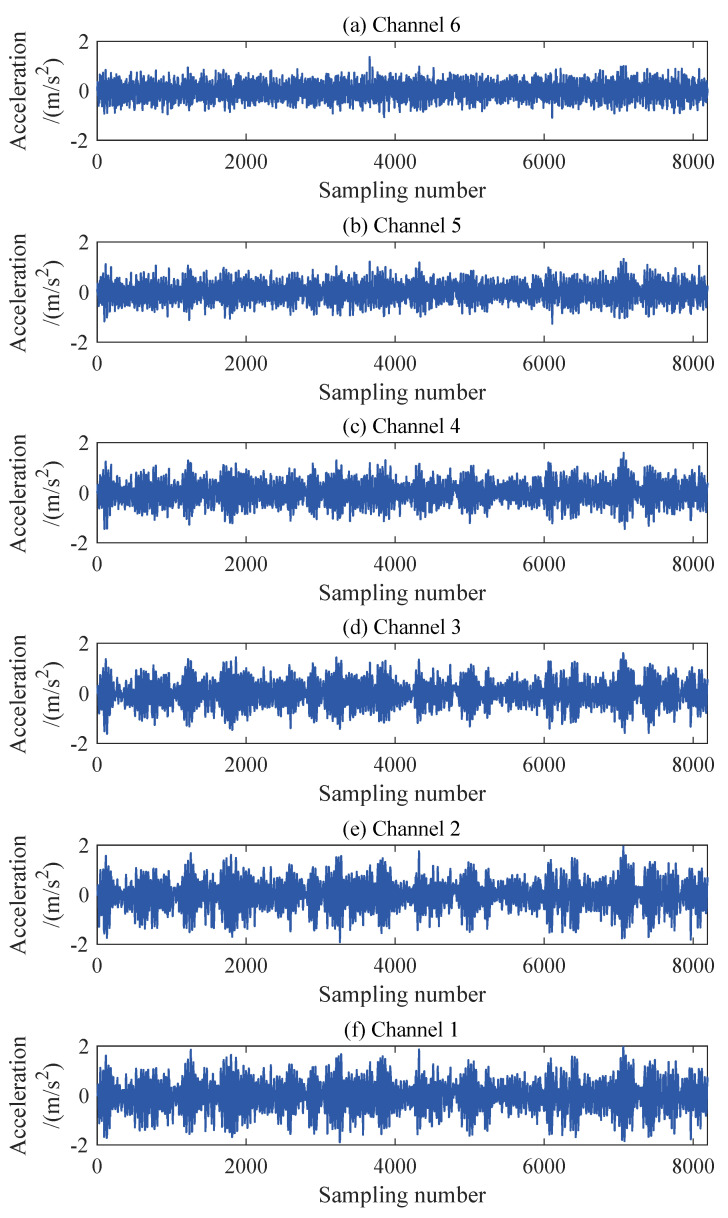
Acceleration response of each channel of a six-story building model.

**Figure 6:**
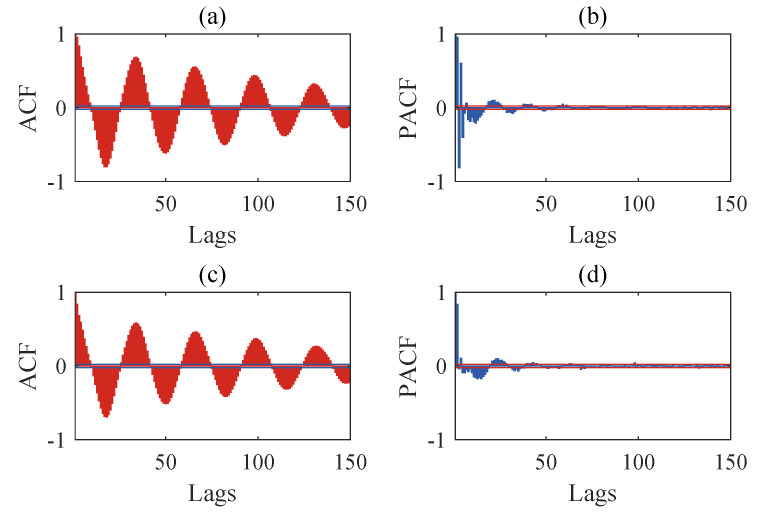
Results of Box-Jenkins analysis: (**a**) autocorrelation function (ACF) for channel 3 in state 1, (**b**) partial autocorrelation function (PACF) for channel 3 in state 1, (**c**) ACF for channel 3 in state 5, and (**d**) PACF for channel 3 in state 5.

**Figure 7:**
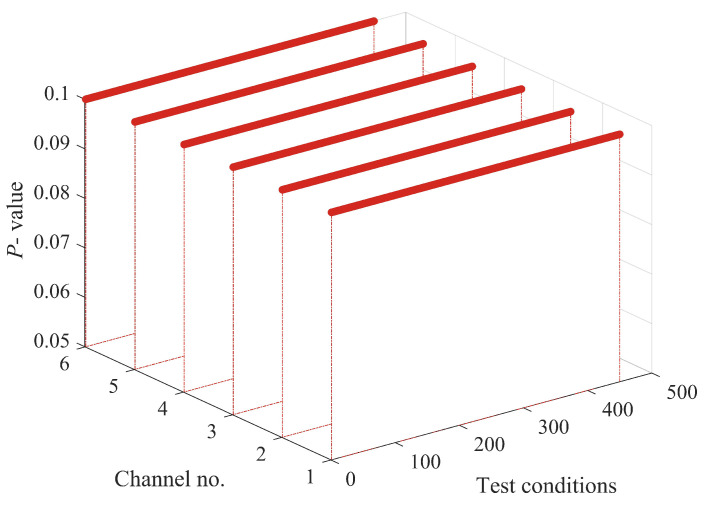
Leybourne-McCabe (LMC) hypothesis test *p*-value for all segments responses of six-story building model at 0.05 significance level.

The structural responses for each state are divided into 50 sets of data, with each set containing 8192 data points of responses. Since there are a total of nine damage conditions, each channel will generate 450 sets of structural responses. Figure 5 illustrates the acceleration responses of various channels in a segment of the baseline state. To validate the applicability of the AR model to the numerical six-story building model, the responses of channel 3 in both state 1 and damage state 5 are utilized for the Box–Jenkins method test. As shown in Figure 6, for both undamaged and damaged states, the autocorrelation function (ACF) of the response of channel 3 exhibits a tailing behavior, while the partial autocorrelation function (PACF) displays a truncation pattern, which partially proves the applicability of the AR model. To further validate the applicability of the AR model to all segmented responses, we conduct the LMC hypothesis test on 6×450 sets of structural responses. Figure 7 shows that the *p*-values of the LMC hypothesis test at the 0.05 significance level for all segmented responses equal 0.1. The AR model is deemed applicable since the *p*-values for all 6×450 sets of structural responses exceed the significance level of 0.05.

Once the suitability of the AR model is established, we initially assess the model orders for channels 1–6 under the baseline condition using the BIC. Specifically, the initial model orders for each channel are determined as follows: 21, 16, 14, 13, 8, and 12. Subsequently, based on these initial model orders, we further refined the model orders using the LBQ test, resulting in the final orders of 21, 19, 28, 19, 20, and 14 for the respective channels. After determining the model order, the AR model is used to fit multiple sets of baseline data, and the average of the model coefficients is calculated as the baseline AR model for each channel. Subsequently, these baseline AR models are used to predict the sensor response in the unknown state, and the residuals are computed as DSFs. Finally, statistical decision analysis of nonlinear damage is performed using classifiers based on KLD, PE, and AAPE.

**Figure 8:**
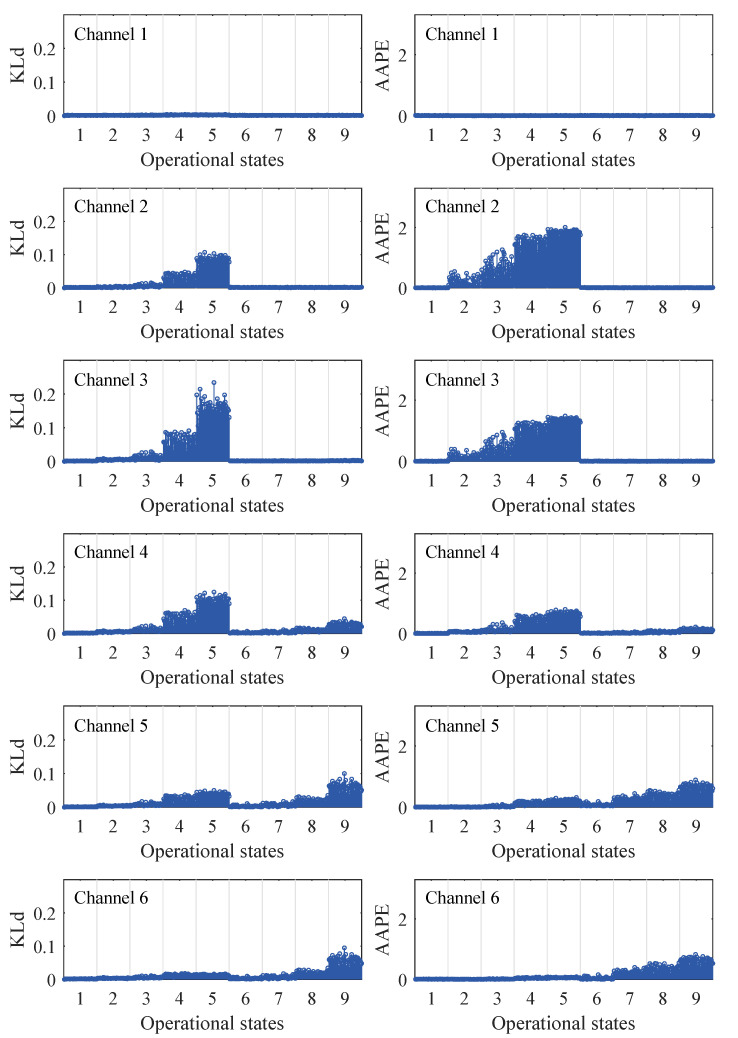
Nonlinear damage diagnostic results of Kullback–Leibler divergence (KLD) and amplitude-aware permutation entropy (AAPE) for different damage states in a six-story building model.

Figure 8 compares the results of nonlinear damage diagnosis for the six-story model based on the KLD and AAPE classifiers. Given a total of nine operating conditions, the classifier for each channel will generate 450 sets of damage indicators, with 50 sets corresponding to each damage state. The results indicate that both KLD and AAPE can detect the occurrence of nonlinear damage, the manifestation of which increases progressively with the severity of the damage. In addition, the KLD and AAPE on the damaged floor slab are significantly higher than the other locations, thus providing information about the location of the damage. It is worth noting that AAPE consistently detects anomalies earlier than KLD in cases of low-level nonlinear damage. For instance, in state 2, minor damage occurs on the third floor. In this case, the AAPE of channels 2 and 3 is noticeably abnormal, whereas the change in KLD is not significant. In state 7, when minor damage occurs on the sixth floor, the AAPEs on channels 5 and 6 similarly detect the occurrence of minor damage before the KLD. These observations all indicate that AAPE exhibits higher sensitivity to low-level nonlinear damage compared to KLD. In addition, similar results can be obtained when compared to PE, which is shown in the experimental case.

To compare the damage localization capabilities of the three classifiers under different levels of damage severity, we calculated the statistical means of each classifier which have been normalized using the following formula [25]:

(12) $$v_{i}^{\prime }=\frac{v_{i}}{\sum_{i=1}^{n}v_{i}}$$

**Figure 9:**
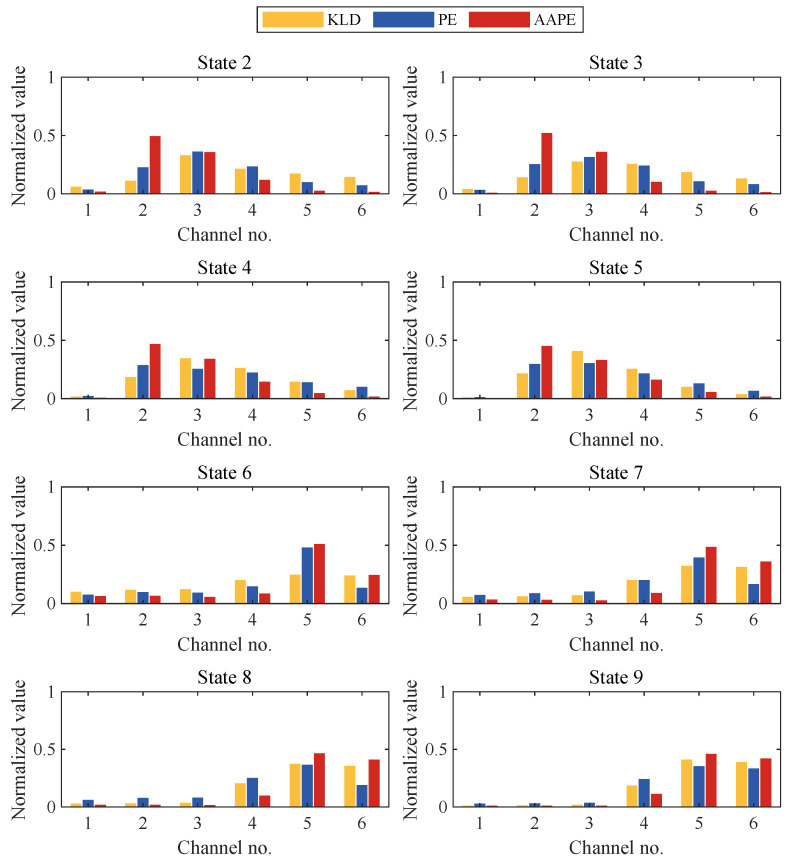
Nonlinear damage localization results of KLD, permutation entropy (PE), and AAPE for different damage states of a six-story building model.

Figure 9 presents the damage diagnosis results of three classifiers under various damage conditions. States 2 to 5 correspond to nonlinear damage on the third floor, where the classifiers should show anomalies in channels 2 and 3. Observations show that all three classifiers indicate damage near channel 3. However, in terms of pinpointing the damage location, AAPE outperforms KLD and PE. Particularly in cases of minor damage (e.g., states 2 and 3), the normalized AAPE values for channels 2 and 3 are close to 0.5, which is significantly higher than KLD and PE. In addition, KLD and PE show more false positives than AAPE in channels 4 and 5. States 6 to 9 correspond to nonlinear damage on the sixth floor. Under more severe damage conditions (e.g., states 8 and 9), the diagnostic results of the three classifiers are similar, all indicating the location of damage at channels 5 and 6. However, under minor damage conditions (e.g., states 6 or 7), PE shows higher normalized values in channel 4 than in channel 6, and KLD’s damage localization in state 6 is not as distinct. In contrast, AAPE’s normalized values at channels 5 and 6 are both close to 0.5, accurately indicating the damage location. Thus, AAPE demonstrates a significant advantage in damage localization, especially under conditions of minor damage.

## 5. Experimental Case

### 5.1. Three-Story Framework Experiment Structure

**Figure 10:**
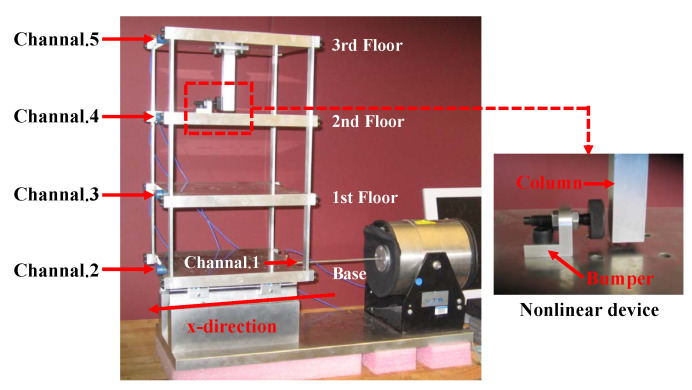
Three-story framework experimental structure.

To further validate the damage diagnostic results of the six-story numerical model, experimental data from the three-story frame at Los Alamos Laboratory are employed in this Section [47]. The frame structure and loading apparatus used in the experiment are shown in Figure 10. The structure is composed of aluminum columns and plates connected by bolts. During the experiment, the model undergoes vibration excitation in the X-direction with a band-limited white noise ranging from 20 to 150 Hz using a vibration exciter. To obtain the acceleration time series data of each aluminum plate in the X-direction, accelerometers are distributed on the centerline of each aluminum plate, with channels 3 to 5 corresponding to the responses of floors 1 to 3, respectively. To simulate structural damage caused by fatigue cracks and loosening bolts, a nonlinear damage source consisting of suspension columns and bumpers is introduced on the structure’s third floor. Channels 4 and 5 correspond to the nonlinear damage location.

The experiment was set up with different damage states, as shown in Table 2. State 1 represented the baseline condition when the structure was undamaged. States 2 to 6 simulated various degrees of nonlinear damage by adjusting the gap between the suspension column and the bumper, which is similar to the parameter *d* in the numerical case. As the gap distance decreased, the degree of nonlinear damage gradually increased. States 7 to 9 simulated nonlinear damage states occurring when mass blocks were added on the ground and first-floor slab. The mass of these blocks was approximately 18.75% of the slab’s mass. Each structural state in the experiment provided structural responses with 409,600 data points collected at a frequency of 320 Hz.

### 5.2. Nonlinear Damage Identification Process and Results

**Figure 11:**
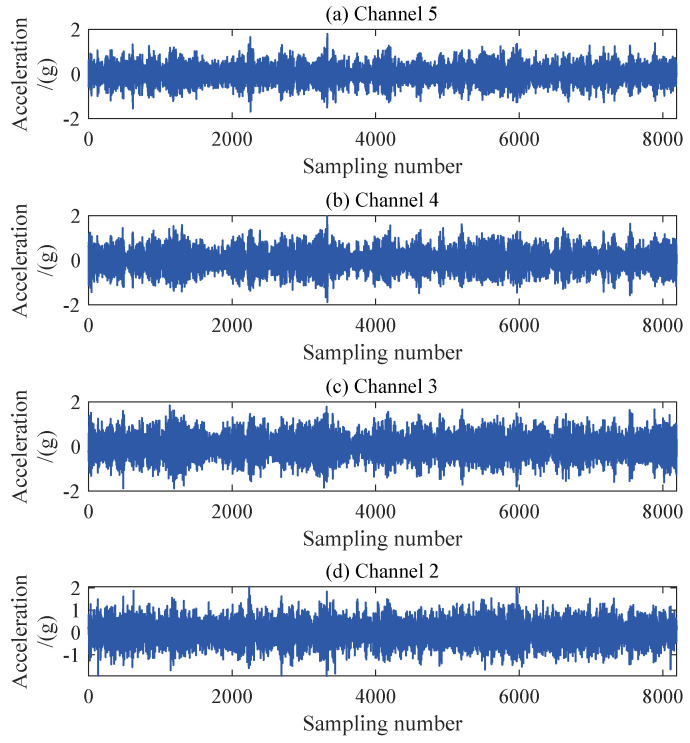
Acceleration response of each floor of three-story frame experimental structure.

**Figure 12:**
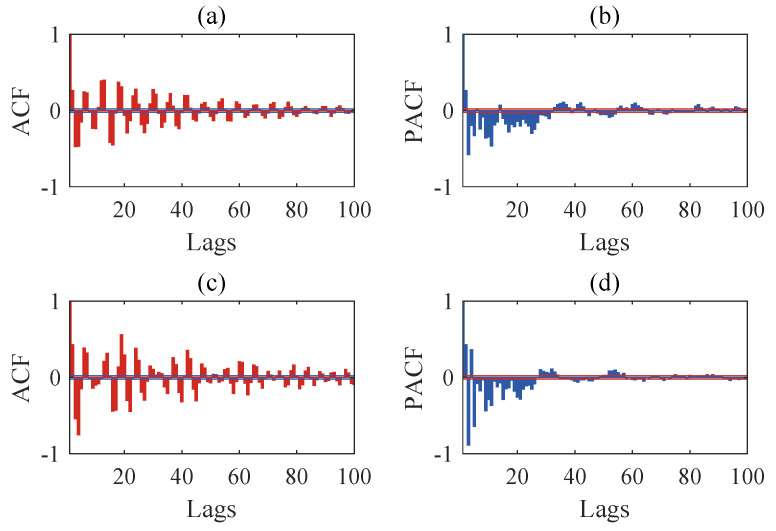
Results of Box-Jenkins analysis: (**a**) ACF for channel 4 in state 1, (**b**) PACF for channel 4 in state 1, (**c**) ACF for channel 4 in state 6, and (**d**) PACF for channel 4 in state 6.

**Figure 13:**
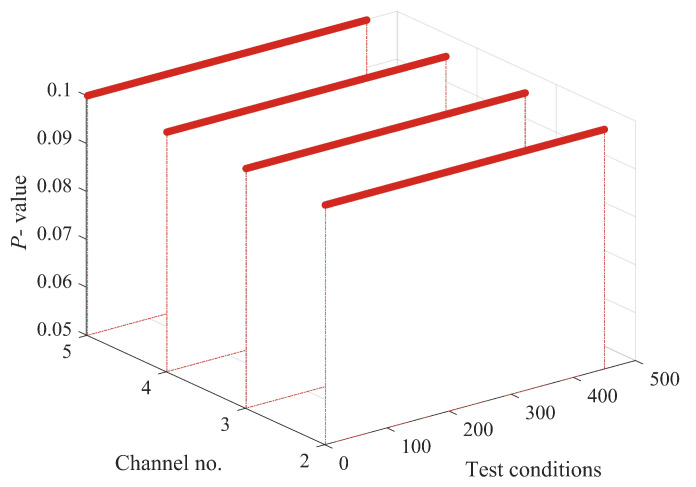
LMC hypothesis test *p*-value for all segmental responses of three-story frame structure at 0.05 level of significance.

The structural responses for each state are divided into 50 groups of data, with each group containing 8192 data points of response. With a total of nine damage conditions, 450 sets of structural responses will be generated for each channel. Figure 11 illustrates the acceleration response of different channels in a set of segments at the baseline state. To validate the applicability of the AR model to the three-story framework structure, the response of channel 4 in baseline state 1 and damage state 6 is utilized for the Box–Jenkins method verification. As shown in Figure 12, under both undamaged and damaged states, the autocorrelation function (ACF) of channel 4 in the three-story framework structure exhibits a tailing behavior, while the partial autocorrelation function (PACF) shows truncation, partially confirming the suitability of the structure’s response for the AR model. To further validate the applicability of segmented responses for all sensor channels to the AR model, we conduct an LMC hypothesis test on 4×450 sets of structural responses. Figure 13 presents the *p*-values of all segmented response LMC hypothesis tests at a significance level of 5%. Since the *p*-values for all 4×450 sets of structural responses exceed the significance level of 0.05, it is demonstrated that the AR model is applicable.

After confirming the applicability of the AR model, we first use the BIC to determine the initial model orders for channels 2–5 under the baseline state, which are 35, 28, 20, and 21, respectively. Then, based on the initial model orders, we improve the model orders using the LBQ test. The final determined model orders are 41, 36, 33, and 35, respectively. Finally, the AR model for each channel is established in the baseline state, and the model residuals under different test conditions are extracted as DSFs. After obtaining each channel’s AR model residual data, nonlinear damage diagnosis is performed using damage classifiers based on KLD, PE, and AAPE.

**Figure 14:**
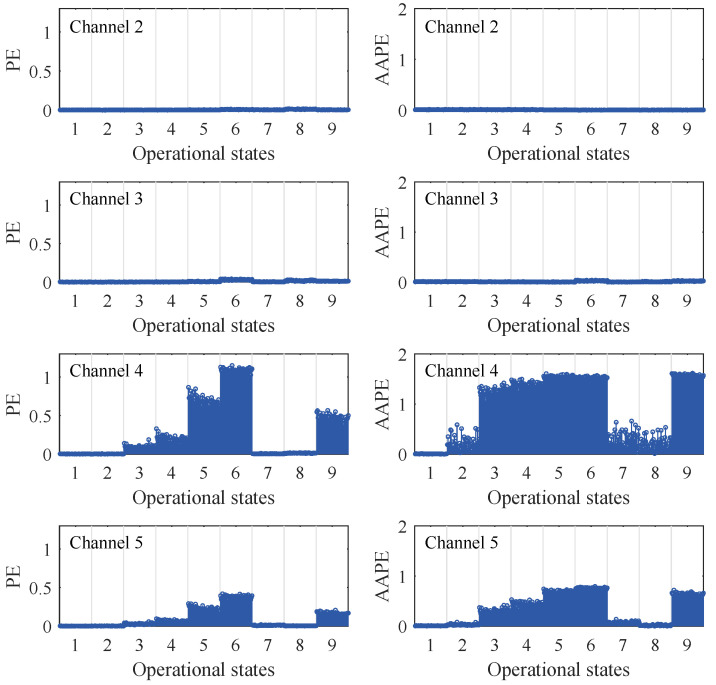
Nonlinear damage diagnostic results of PE and AAPE for different damage states in the three-story framework structure.

Figure 14 illustrates the damage diagnostic results of each channel in terms of PE and AAPE. Considering a total of nine damage scenarios, including baseline, the classifier will generate 450 sets of damage indicators per channel, with 50 groups corresponding to each damage state. The results indicate that, with the occurrence of damage, both PE and AAPE for channel 4 and channel 5 exceed the baseline level, while the entropy values for channel 2 and channel 3 exhibit little change. This reveals that both PE and AAPE are effective in detecting the occurrence of nonlinear damage and can reflect its location. Furthermore, as the degree of nonlinear damage increases, PE and AAPE gradually increase. This indicates that both can qualitatively reflect changes in the degree of nonlinear damage. It is worth noting that, similar to the numerical structural case, AAPE always detects the occurrence of damage earlier than PE when minor damage occurs. For instance, in state 2, a slight nonlinear damage occurs on the third floor. At this point, the abnormality is detected by AAPE in channel 4, while PE in channel 4 does not detect any abnormalities. In addition, while PE detects minor nonlinear damage in state 3, which is more severe than in state 2, AAPE is even more sensitive to this type of minor damage. These phenomena are consistent with the results of the numerical structure analysis and demonstrate the advantages of AAPE in the early diagnosis of nonlinear damage.

**Figure 15:**
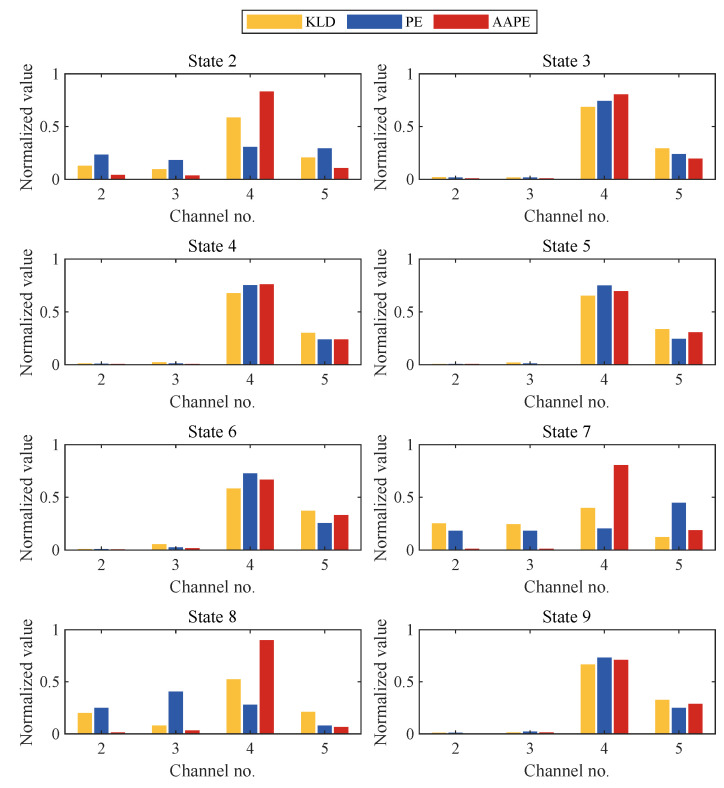
Nonlinear damage localization results of KLD, PE, and AAPE for different damage states of the three-story framework structure.

Figure 15 presents the damage localization results of different classifiers. For severe damage levels (e.g., states 3, 4, 5, and 6), the normalized values of all three damage classifiers indicate anomalies near channels 4 and 5, aligning closely with the damage location. However, under minor damage conditions (e.g., state 2), the normalized values of KLD and PE at channels 4 and 5 significantly decrease, with PE even exhibiting anomalies at the undamaged channels 2 and 3. Furthermore, in cases involving changes in the quality of the structural foundation or the first floor (e.g., states 7 and 8), the PE is no longer able to correctly indicate the location of the damage, and the KLD’s indication of the location of the damage is even less distinct. Unlike PE and KLD, in the case of minor damage (e.g., state 2) and minor damage involving changes in structural quality (e.g., states 7 and 8), the normalized AAPE value for channel 4 near the damage location exceeds 0.5 and is even close to the maximum value 1, which allows for the correct localization of the damage. Hence, mirroring the findings from numerical simulations, AAPE evidently holds an edge in the accurate localization of minor structural damage.

## 6. Discussion

During the operation of civil structures, low-level nonlinear damage is often a precursor to progressive structural deterioration. Successfully detecting and localizing such minor damage can significantly enhance the efficiency of SHM and is more conducive to structural maintenance and rehabilitation. Therefore, damage diagnostic methods should have a high sensitivity to minor damage. This paper compares the ability of the proposed AAPE damage classifier with a state-of-the-art damage classifier based on the residuals of AR models in detecting and localizing nonlinear damage at different severity of damage. The research reveals that the damage classifier based on AAPE exhibits a noticeable advantage in detecting low-level nonlinear damage compared to PE and KLD. This could be attributed to:

- Nonlinear damage causes the AR model residuals to contain complex dynamical features such as harmonics or intermodulation distortion. AAPE can measure the complexity of the data, which is useful for identifying potential nonlinear behavior;
- Nonlinear damage leads to a gradual increase in the residual amplitude of the AR model. The AAPE captures changes in the amplitude difference and mean value of adjacent samples of the residual signal, which is more sensitive to the amplitude characteristics of the signal.

Although this paper only demonstrates the superiority of AAPE based on the residuals of AR models for minor damage, AAPE is equally applicable to other time series models, such as the AR-ARX [22,24] and ARX [49,50] models, making it a potential tool for detecting nonlinear damage in structures.

## 7. Conclusions

This study presents a data-driven nonlinear damage diagnosis method using AR models and AAPE. Although AR models have been widely used in diagnosing structural nonlinear damage, most current methods rely on the statistical characteristics of the model residuals. The main contribution of this study is to introduce AAPE as a novel damage classifier for nonlinear damage detection based on AR model residuals. Compared with established methods, AAPE not only extracts damage information from the complexity of the residuals but also exhibits a high sensitivity to changes in the amplitude of the model residuals. Numerical and experimental structures validate the effectiveness and superiority of the proposed method. The main conclusions can be summarized as follows:

1. The proposed approach applies to diagnosing structural nonlinear damage caused by fatigue cracks. The method has a high sensitivity to minor nonlinear damage and good robustness to measurement noise. Therefore, the method can be used for early damage diagnosis at low nonlinear damage levels;
2. The proposed method has the ability to accurately localize damage. In the vicinity of the damaged floor, the damage classifiers are significantly higher than those of other floors. The method also provides accurate information about the location of the damage, even when minor damage scenarios are involved;
3. The proposed method is applicable to parallel and distributed sensor systems with unsupervised learning. It can effectively detect and localize nonlinear damage sources even in the presence of linear variations in structural mass, which is beneficial for practical applications;
4. Only univariate nonlinear damage classifiers are compared and analyzed. Future research will consider a hybrid distance method using AAPE and compare it with existing methods in a more realistic structure;
5. This paper focuses on structural scenarios with a single source of damage, whereas multiple sources of damage may exist in real structures. The challenges posed by multiple sources of damage and different nonlinear damage types will be considered in subsequent studies.

## Figures and Tables

**Table 1:** Operational states of the numerical model of the six-story building.

States	Description
State 1	Undamaged baseline condition
State 2	Damage in the third story, *d* = 0.2 mm
State 3	Damage in the third story, *d* = 0.12 mm
State 4	Damage in the third story, *d* = 0.08 mm
State 5	Damage in the third story, *d* = 0.05 mm
State 6	Damage in the sixth story, *d* = 0.08 mm
State 7	Damage in the sixth story, *d* = 0.03 mm
State 8	Damage in the sixth story, *d* = 0.023 mm
State 9	Damage in the sixth story, *d* = 0.015 mm

**Table 2:** Operational states of the three-story frame structure.

States	Description
State 1	Undamaged baseline condition
State 2	Gap = 0.20 mm
State 3	Gap = 0.15 mm
State 4	Gap = 0.13 mm
State 5	Gap = 0.10 mm
State 6	Gap = 0.05 mm
State 7	Base adds 1.2 kg mass and 0.20 mm gap
State 8	1st-floor slab add 1.2 kg mass with 0.20 mm gap
State 9	1st-floor slab add 1.2 kg mass with 0.10 mm gap

## Data Availability

Data are contained within the article.
